# Cernunnos/Xlf Deficiency Results in Suboptimal V(D)J Recombination and Impaired Lymphoid Development in Mice

**DOI:** 10.3389/fimmu.2019.00443

**Published:** 2019-03-14

**Authors:** Benoit Roch, Vincent Abramowski, Julie Chaumeil, Jean-Pierre de Villartay

**Affiliations:** ^1^Laboratory “Genome Dynamics in the Immune System”, INSERM UMR1163, Paris, France; ^2^Institut Imagine, Université Paris Descartes Sorbonne Paris Cité, Paris, France; ^3^Institut Cochin, INSERM U1016, CNRS UMR8104, Université Paris-Descartes, Paris, France

**Keywords:** lymphoid development, V(D)J recombination, positive selection, DNA repair, TCR repertoire

## Abstract

Xlf/Cernunnos is unique among the core factors of the non-homologous end joining (NHEJ) DNA double strand breaks (DSBs) repair pathway, in the sense that it is not essential for V(D)J recombination *in vivo* and *in vitro*. Unlike other NHEJ deficient mice showing a SCID phenotype, *Xlf* ^−/−^ mice present a unique immune phenotype with a moderate B- and T-cell lymphopenia, a decreased cellularity in the thymus, and a characteristic TCRα repertoire bias associated with the P53-dependent apoptosis of CD4+CD8+ DP thymocytes. Here, we thoroughly analyzed *Xlf* ^−/−^ mice immune phenotype and showed that it is specifically related to the DP stage but independent of the MHC-driven antigen presentation and T-cell activation during positive selection. Instead, we show that V(D)J recombination is subefficient in *Xlf* ^−/−^ mice *in vivo*, exemplified by the presence of unrepaired DSBs in the thymus. This results in a moderate developmental delay of both B- and T-lymphocytes at key V(D)J recombination dependent stages. Furthermore, subefficient V(D)J recombination waves are accumulating during TCRα rearrangement, causing the typical TCRα repertoire bias with loss of distal Vα and Jα rearrangements.

## Introduction

All living organisms are exposed to DNA double strand breaks (DSBs), described as the most toxic type of DNA damage. DSBs result from either external genotoxic stresses or endogenous physiological processes (1), such as V(D)J recombination and Class Switch Recombination during T- and B-lymphocytes development and maturation (2), meiotic recombination, or during RNA Polymerase II-driven transcription of early response genes following cell activation or heat shock (3, 4). Exogenous and physiological DSBs are repaired by either homologous recombination (HR) or the non-homologous end-joining (NHEJ) pathway, the later proceeding via the direct ligation of DNA ends (5). Briefly, the NHEJ pathway is composed of core factors, Ku70-Ku80 and the DNA-dependent protein kinase-catalytic subunit (DNA-PKcs) for DSBs recognition and stabilization, Artemis endo/exonuclease and Terminal-deoxynucleotydyl-Transferase (TdT), DNA polymerases μ and λ for DNA ends processing if necessary, and the Xlf-XRCC4-DNA Ligase IV complex for the final ligation step. In this last step, Xlf, and XRCC4 homodimers form a long polymeric filament tethering DNA broken ends, thus creating a “DNA repair synapse” (6–8). XRCC4 also stabilizes and activates DNA Ligase IV, which ensures the final repair of aligned DNA ends (9).

In T- and B-cell precursors in the thymus and bone marrow, respectively, variable domains of antigen receptors are somatically rearranged from V, D, and J gene loci by V(D)J recombination. Two steps of thymocyte maturation are closely associated with V(D)J recombination: first, the “TCRβ selection” at the CD4-CD8- Double Negative (DN) stage upon successful rearrangement of the *Tcrb* locus (10) and second, the “positive selection” at the CD4+CD8+ Double Positive (DP) stage following productive rearrangement of the *Tcra* locus (11). The lymphoid-specific complex RAG1/RAG2 initiates V(D)J recombination by introducing DSBs within Recombination Signal Sequences (RSS) flanking V, D, and J coding segments. RAG1/2 complexes remain bound to DSB ends as the post cleavage complex (PCC) to stabilize broken DNA ends prior to their repair by NHEJ (12). NHEJ function is critical for T- and B-lymphocyte development. Indeed it is the sole DNA repair pathway to cope with RAG1/2 generated DSBs, which occur during G0/G1 of the cell cycle. Loss of function of core NHEJ factors results in severe combined immunodeficiency (SCID) conditions in both humans and mice, owing to aborted V(D)J recombination (13). XRCC4 or DNA Ligase IV gene inactivation in mice results in SCID phenotype and embryonic lethality secondary to massive apoptosis of post-mitotic neurons (14, 15). Furthermore, defects in NHEJ result in genetic instability, and unrepaired DSBs produced during V(D)J recombination may lead to T and Pro-B cell lymphomas with *Tcra/d* or *Igh* genes translocation, respectively (16). Pro-B cell lymphomas are hallmarks of NHEJ defects in *Trp53*^−/−^ mice, owing to inefficient DNA repair during V(D)J recombination. Thus, the P53 pathway plays a key role in the thymus by preventing genomic instability in case of aborted V(D)J recombination and DSBs left unrepaired (12, 17). Though the P53 pathway prevents genomic instability through apoptosis of thymocytes with aborted V(D)J recombination, it is noteworthy that it is not involved in the apoptotic “death by neglect” of thymocytes with inadequate TCRαβ during positive selection (18, 19).

Xlf (also known as Cernunnos) is a unique NHEJ core factor in the sense that, although it is essential for the repair of genotoxic DSBs from various origins as shown by the extreme ionizing radiation sensitivity of Xlf deficient cells (20, 21), it is largely dispensable for V(D)J recombination. Indeed V(D)J recombination yields on endogenous *Ig*κ loci and exogenous substrates are normal *in vitro* in v-Abl transformed *Xlf* ^−/−^ Pro-B cells (22, 23). *Xlf* ^−/−^ mice show a very modest immunodeficiency with decreased lymphocyte counts in blood and a loss of cellularity in the thymus (22, 24). Furthermore, *Xlf* ^−/−^ *Trp53*^−/−^ double *Knock-Out* (DKO) mice do not develop Pro-B cell lymphomas commonly seen in other *NHEJ-Trp53* DKO conditions (22), further attesting for the absence of a major V(D)J recombination defect in these mice, and the thymic cellularity is even partly rescued in the *Trp53*^−/−^ background (24). We previously proposed that Xlf participates in a two-tier safeguard mechanism during V(D)J recombination to avoid genetic instability (23, 24). Many DNA repair factors, PAXX, ATM, H2A.X, and MRI, are functionally redundant with Xlf during V(D)J recombination, as revealed by the complete defect in V(D)J recombination *in vitro*, and *in vivo* in doubly deficient situations (25–27). This functional redundancy is mediated, at least in part, through the C-terminus region of RAG2 (23). One key feature of Xlf deficient patients and mice is a remarkable TCRα repertoire bias with the loss of distal VαJα rearrangements (24), a characteristic first revealed in the context of a reduced thymocyte lifespan such as in RORγT and TCF-1 KOs mice (28, 29). *Xlf* ^−/−^ immune phenotype in mice is indeed associated with a decreased thymocyte lifespan owing to chronic activation of the P53 pathway and apoptosis *ex vivo* (24). More recently, we identified a similar TCRα repertoire skewing in various human conditions of DNA repair deficiencies or hypomorphic RAG1/2 mutations (30).

At least two non-exclusive hypotheses can be raised to account for the immune phenotype of *Xlf* ^−/−^ mice in the context of an apparent normal V(D)J recombination. On one hand, one may propose that subefficient V(D)J recombination is associated with the persistence of DSBs and the chronic activation of P53. We used dedicated and sensitive markers to reveal possible persistence of unrepaired DSBs in thymocytes during V(D)J recombination. On the other hand, one alternative hypothesis could be that Xlf deficiency leads to a premature thymocyte death during positive selection at the DP stage, thus interfering with the ongoing multiple TCRα rearrangements at this stage. As positive selection strictly relies on antigen presentation by MHC class I and class II molecules, we introduced the *Xlf* ^−/−^ onto *MHC* deficient background to analyze this second proposition.

## Results

### *Xlf*^−/−^ Thymus Loss of Cellularity Is Not Related to Positive Selection at the DP Stage

**Figure 1:**
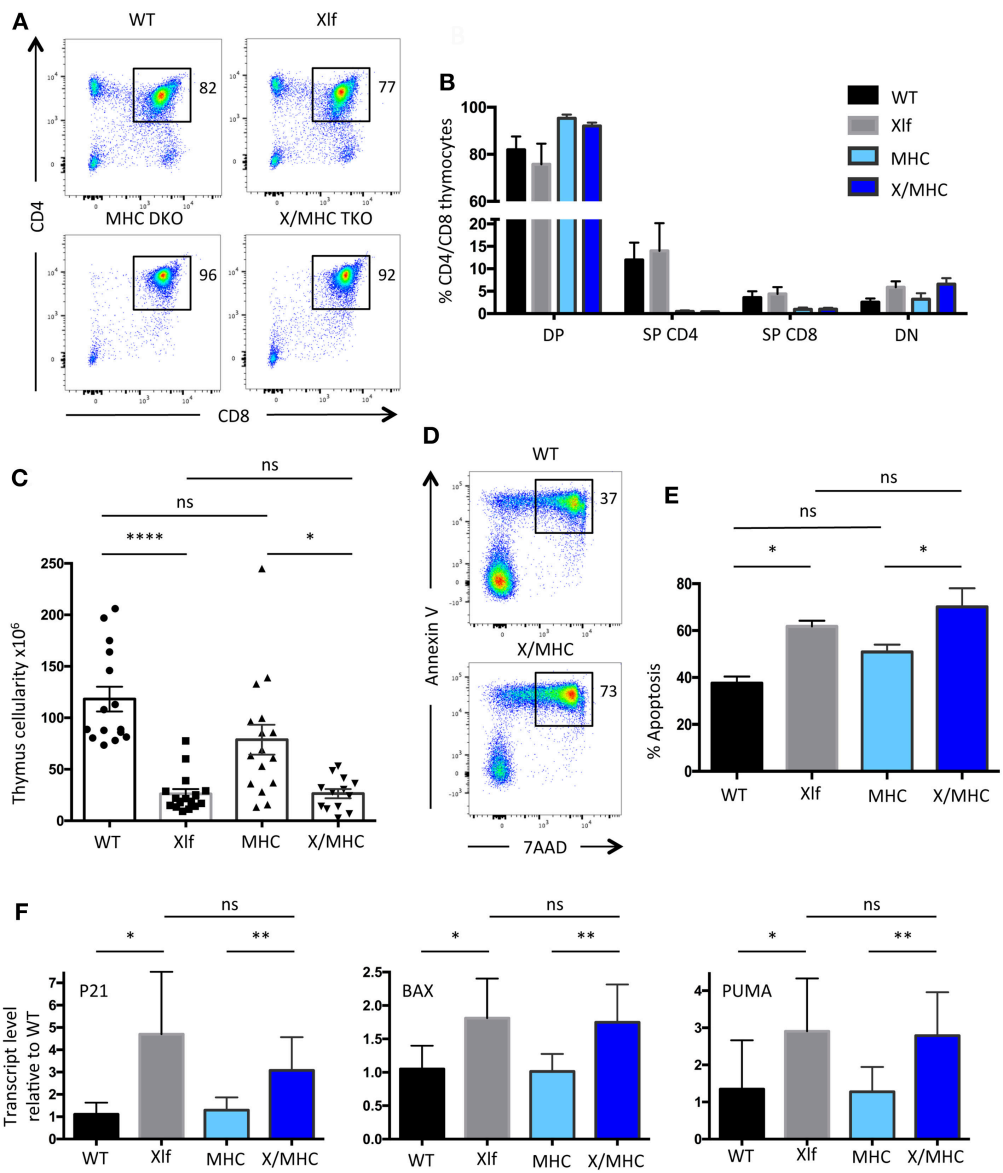
*Xlf*^−/−^ immune phenotype is not related to positive selection at the DP stage in the thymus. **(A)** Immunostaining of CD4/CD8 in thymii of WT, Xlf^−/−^ (Xlf), MHC class I^−/−^ MHC class II^−/−^ (MHC DKO), Xlf^−/−^ MHC class I^−/−^ MHC class II^−/−^ (X/MHC TKO) mice. **(B)** Quantification of CD4/CD8 thymic populations. **(C)** Total thymocyte numbers. **(D)** Thymocyte apoptosis analyzed by Annexin V/7AAD staining following 20 h of culture. **(E)** Quantification of thymocyte apoptosis after 20 h of culture. **(F)** Quantitative RT-PCR analysis of P53 target genes P21, BAX, and PUMA in thymocyte freshly extracted RNA. P-values were calculated using non-parametric Kruskall-Wallis with Dunn's correction tests **(C,E)** and non-parametric Mann-Whitney tests **(F)**.

Our previous analyses of the *Xlf* ^−/−^ mice established that the decrease in thymocyte viability mainly occurs at the CD4+CD8+ Double-Positive (DP) stage, resulting in the characteristic TCRα repertoire skewing (24). DP stage is the time of thymocyte positive selection, which arises through MHC dependent TCR activation (11). As cellular activation has been associated with the occurrence of DSBs in various settings (3, 4) we wished to evaluate the possible impact of positive selection in the phenotype of *Xlf* ^−/−^ mice. We prevented DP thymocyte activation and positive selection by crossing *Xlf* ^−/−^ mice with *MHC class I*^−/−^ *MHC class II*^−/−^ double KO (*MHC* DKO) mice (31). T-lymphocyte development was arrested at the DP stage in the thymus of both *MHC* DKO and *Xlf* ^−/−^ *MHC class I*^−/−^ *MHC class II*^−/−^ Triple KO (*Xlf/MHC* TKO) mice, with a sharp decrease in CD4 and CD8 single positive thymocytes as expected in the absence of positive selection (Figures 1A,B). Indeed, all DP thymocytes were CD69 negative in both settings, attesting for the lack of TCR mediated activation (data not shown). The thymocyte count, which was significantly reduced in *Xlf* ^−/−^ mice (26.2 × 10^6^ ± 4.6 SEM in *Xlf* ^−/−^ vs. 118 × 10^6^ ± 12.0 SEM in WT, *p* < 0.0001) (Figure 1C) as previously described (22, 24, 27), was not rescued in *Xlf/MHC* TKO mice (26.4 × 10^6^ ± 4.4 SEM) and remained statistically decreased as compared to *MHC* DKO (78.8 × 10^6^ ± 14.6 SEM, *p* = 0.02) (Figure 1C). The absolute number of CD4-CD8- Double-Negative (DN) thymocytes was unchanged in *Xlf* ^−/−^ and *Xlf/MHC* TKO as compared to WT or *MHC* DKO littermates (respectively 1.80 × 10^6^ ± 0.33 SEM and 2.18 × 10^6^ ± 0.31 SEM vs. 2.70 × 10^6^ ± 0.27 SEM and 3.60 × 10^6^ ± 0.53 DN thymocytes). Furthermore, the *MHC* null background did not rescue the *ex vivo* thymocyte apoptosis caused by Xlf deficiency after 20 h of culture (24) as measured by dual staining 7AAD and Annexin V (61.8% ± 2.8 SEM in *Xlf* ^−/−^ and 70.1% ± 1.9 SEM in *Xlf/MHC* TKO *p* = 0.015 vs. 37.6% ± 2.8 SEM in WT and 50.9% ± 3.1 SEM in *MHC* DKO) (Figures 1D,E). Lastly, the chronic activation of the P53 pathway in *Xlf* ^−/−^ thymocytes, revealed by the induced expression of P53 target genes *P21, PUMA*, and *BAX* (24), was not reversed by the MHC deficient background in *Xlf/MHC* TKO thymocytes (Figure 1F).

Altogether, these results suggest that the process of positive selection at the DP stage is not the cause of the P53 chronic activation and resulting cell death and decreased thymocyte cellularity caused by the Xlf loss of function.

### *Xlf*^−/−^ TCRα Repertoire Bias Is Independent of Antigen Presentation

**Figure 2:**
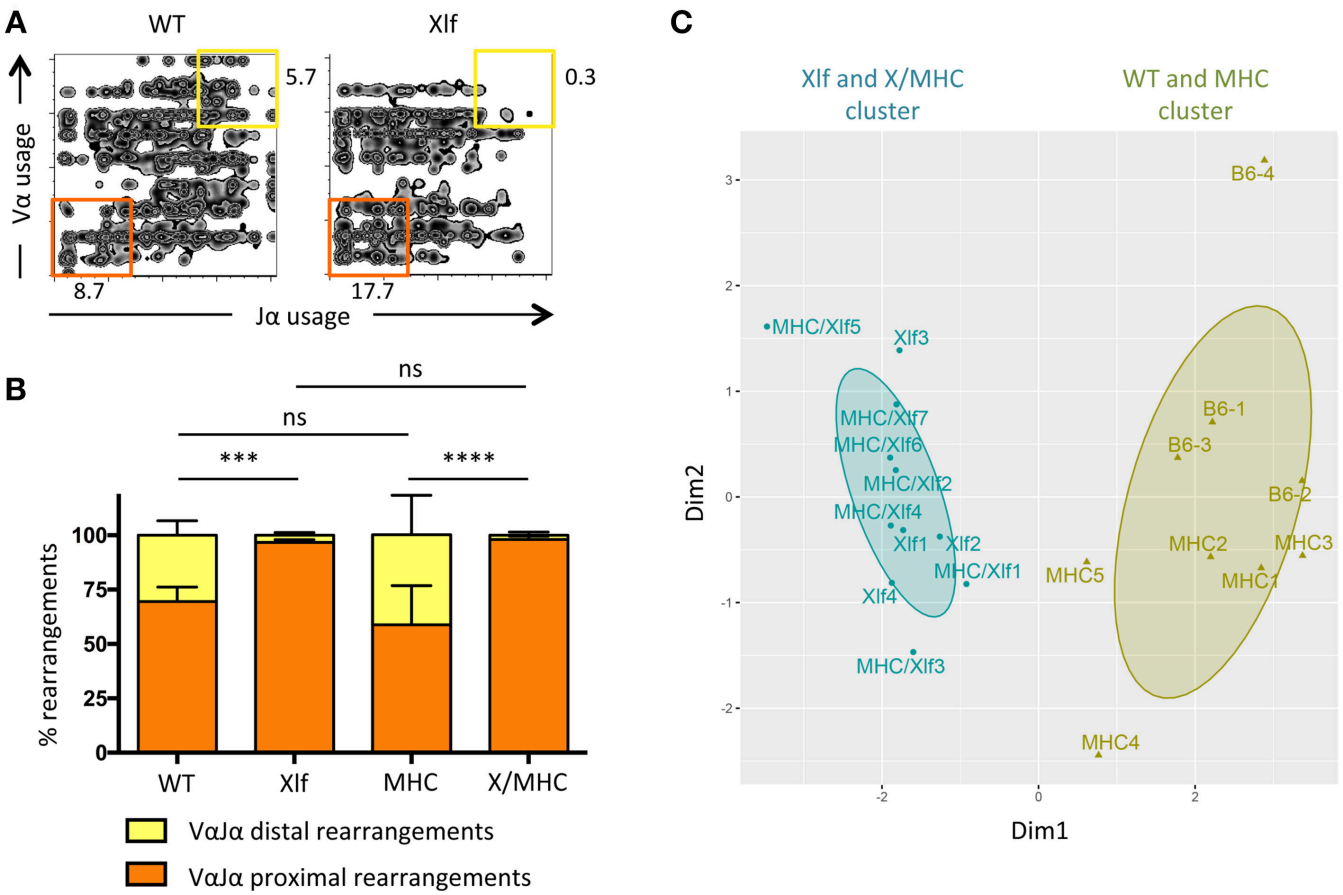
*Xlf*^−/−^ TCRα repertoire bias is independent of MHC driven antigen presentation during thymic development. **(A)** Illustrative 2-dimension plot representation of TCRα repertoire from whole thymus. Each node represents the association between one TRAV and one TRAJ segment as determined by TCRα transcript sequencing. **(B)** Relative usage of proximal and distal TRAV-TRAJ rearrangements. *P*-values were calculated using non-parametric Mann-Whitney tests. **(C)** Illustrative representation of Principal Component Analysis/unsupervised Hierarchical Clustering analysis (PCA/HC) analysis of TRAV and TRAJ usage in thymus according to PROMIDISα (30).

Having established that the MHC class I class II deficiency did not have an appreciable impact on cell death and thymocyte lifespan of Xlf deficient thymocytes, we wished to analyze the consequences on the generation of the TCRα repertoire. The V(D)J recombination of the TCRα locus is unique compared to the other TCR and Ig loci, in the sense that multiple waves of TCRα rearrangement occur until the expressed TCRαβ can recognize an antigen presented by MHC molecules for positive selection (32). Thymocyte viability directly affects the number of successive VαJα rearrangements. Decreased DP thymocyte lifespan (such as in the RORγT and TCF-1 deficient mice) leads to the loss of distal VαJα rearrangements and deficit of iNKT cells, which require the specific distal Vα14Jα18 rearrangement, in patients and mice (28, 33, 34). On the other hand, extending thymocyte lifespan [through Bcl-x(L) transgene for example] leads to the opposite effect; namely an over-representation of distal rearrangements (28, 29). The TCRα repertoire is altered in Xlf deficient mice and patients possibly as a consequence of DP thymocyte lifespan decrease (24, 35). We analyzed the impact of MHC deficiency on the TCRα repertoire through 5′ RACE RT-PCR followed by deep sequencing of mTRAV–mTRAJ junctions from whole thymocytes (Figure 2). The skewed TCRα repertoire, with strongly decreased usage of distal VαJα rearrangements and the concomitant increased usage of proximal VαJα rearrangements (Figures 2A,B), which is characteristic of Xlf deficiency, was not rescued by the MHC deficient background. Indeed, principal component analysis (PCA) and unsupervised hierarchical clustering (HC) analysis using PROMIDISα biomarker (30), which takes into account several parameters of TCR Vα and Jα usage, grouped *Xlf* ^−/−^ and *Xlf/MHC* TKO thymocytes in the same cluster, distinct from that of WT and *MHC* DKO thymocytes (Figure 2C).

From this first set of analyses, we conclude that putative unrepaired DSBs that could occur in the course of T-cell activation during positive selection of DP thymocytes does not account for the decreased thymocyte viability and the skewed TCRα repertoire in *Xlf* ^−/−^ mice, since none of these phenotypical traits were rescued in the absence of positive selection subsequent to *MHC class I* and *class II* genes inactivation. Nevertheless, *Xlf* ^−/−^ thymocyte loss of viability is connected to the DP stage, when V(D)J recombination at the *Tcra* loci is taking place. Interestingly, biased TCRα repertoire with a similar loss of distal VαJα rearrangements has recently been described in various human conditions characterized by hypomorphic mutations in known factors of the V(D)J recombination machinery (i.e., *RAG1, Artemis, DNA Ligase IV, Cernunnos/Xlf*, *PRKDC* genes) (30). These hypomorphic mutations, which result in subefficient TCRα rearrangement waves, ultimately translate into a skewed TCRα repertoire when the repertoire of other *Tcr* loci appears grossly unaffected. This raises the possibility that the biased TCRα repertoire in *Xlf* ^−/−^ mice may not be the consequence of the decreased thymocyte viability *per se* (such as in RORγT deficient mice) but secondary to a subefficient V(D)J recombination activity (such as in RAG1 hypomorphic conditions).

### DNA Repair Defect During TCRα Rearrangements in *Xlf*^−/−^ DP Thymocytes

**Figure 3:**
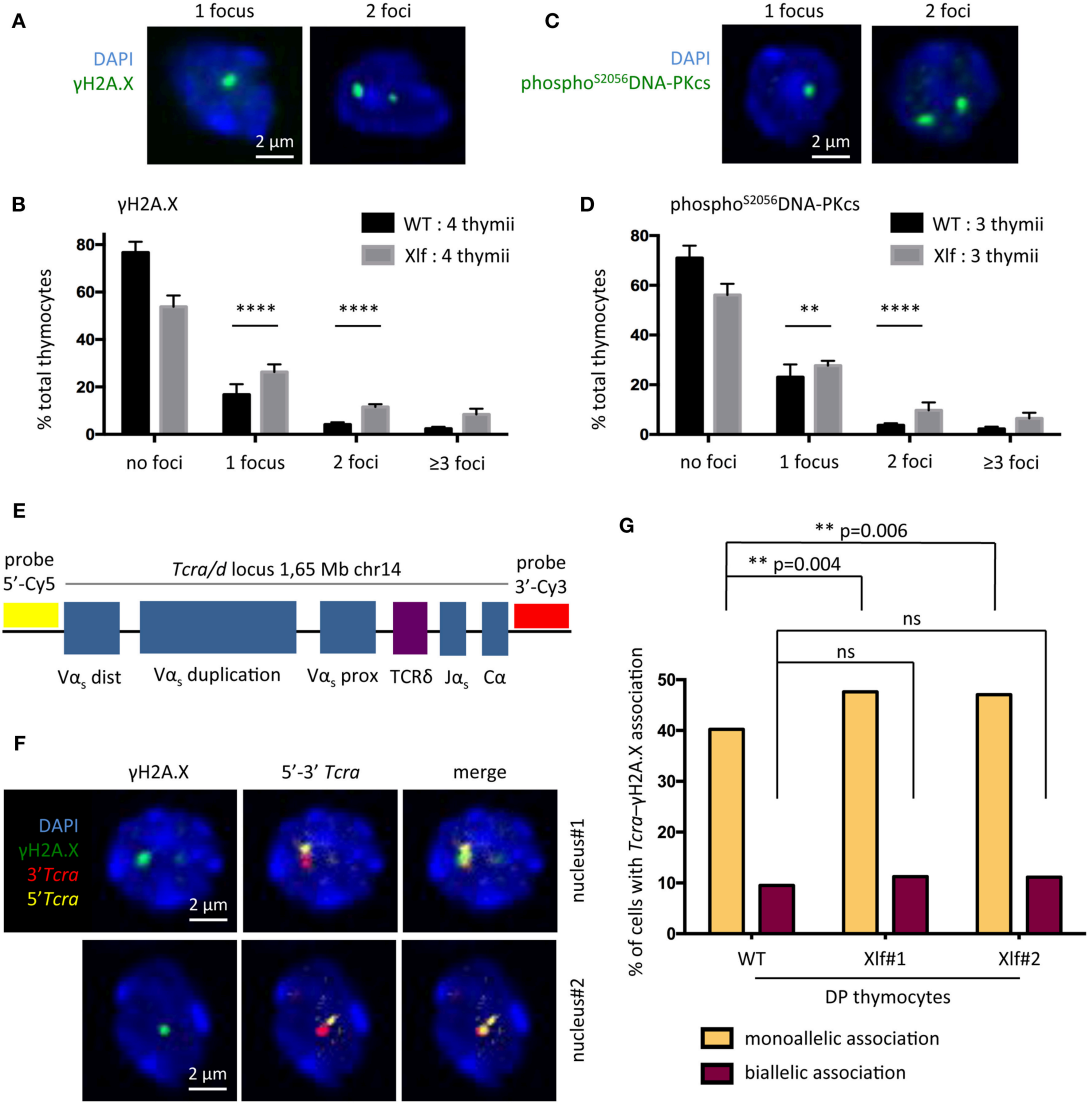
DNA repair defect during TCRα rearrangement in *Xlf*^−/−^ thymocytes. **(A)** γH2A.X DNA repair foci 3-dimensional immunofluorescence in total thymocytes. **(B)** Quantification of thymocyte nuclei presenting 0, 1, 2 or more than 3 γH2A.X foci. From 200 to 500 nuclei per thymus were scored. **(C)** Phospho^S2056^DNA-PKcs DNA repair foci 3-dimensional immunofluorescence of in total thymocytes. **(D)** Quantification of thymocyte nuclei presenting 0, 1, 2 or more than 3 phospho^S2056^DNA-PKcs foci. From 200 to 500 nuclei per thymus were scored. **(E)** Schematic representation of the *Tcra/d* locus in mice and positions of the probes used for DNA-FISH. RP23-255N13 and RP23-304L21 BAC probes label the 3′ and 5′ edges of the *Tcra/d* locus, respectively. **(F)** 3-dimensional Immuno-DNA-FISH analysis of *TCR*α rearrangement in sorted CD4+CD8+ DP thymocytes in 2 independent nuclei. A γH2A.X focus associated with *Tcra* locus indicates an ongoing TCRα rearrangement. **(G)** Quantification of CD4+CD8+ DP thymocyte nuclei displaying 1 or 2 *Tcra*-γH2A.X association. 1 thymus from a WT mouse and 2 thymii from Xlf^−/−^ mice were analyzed. Around 550 nuclei per thymus were scored. *P*-values were calculated using two-tail Fisher's exact test **(B,D,G)**.

To test the possibility that subefficient V(D)J recombination resulting in unrepaired RAG1/2 induced DSBs may be responsible for the phenotype of *Xlf* ^−/−^ thymocytes we first analyzed γH2A.X and phospho^S2056^DNA-PKcs DNA repair foci by immunofluorescence in total thymocytes, as surrogate markers of unrepaired DSBs. Quantification of γH2A.X (Figures 3A,B) and phospho^S2056^DNAPKcs foci (Figures 3C,D) showed a significant increase in cells with 1 or 2 DNA repair foci in *Xlf* ^−/−^ vs. WT thymocytes, with an increase of 16.76 to 26.31% (*p* < 0.0001) of cells with 1 focus and 4.18 to 11.55% (*p* < 0.0001) of cells with 2 foci for γH2A.X and 23.05 to 27.70% (*p* = 0.006) of cells with 1 focus and 3.73 to 9.72% (*p* < 0.0001) of cells with 2 foci for phospho^S2056^DNAPKcs foci in *Xlf* ^−/−^ vs. WT thymocytes. The frequency of thymocytes with more than 3 γH2A.X or phospho^S2056^DNAPKcs foci, which may represent dying cells, also increased from 2.4 to 8.4% (*p* < 0.0001) and 2.3 to 6.5% (*p* < 0.0001) for γH2A.X and phospho^S2056^DNA-PKcs respectively in WT vs. *Xlf* ^−/−^ thymocytes, highlighting the overall thymocyte decreased viability. To analyze whether the increased γH2A.X foci indeed corresponded to unrepaired DSBs occurring during V(D)J recombination at *Tcra* loci at the DP stage in *Xlf*^−/−^ condition, we sorted CD4+CD8+ DP thymocytes from 6 to 9 weeks mice, and performed *Tcra*-γH2A.X association analysis by immuno-DNA fluorescence *in situ* hybridization (FISH). We used an antibody against γH2A.X as a read-out for random and RAG mediated DSBs (36) in combination with two DNA probes that hybridize to the 5′ and 3′ ends of the full length *Tcra/d* locus, respectively (37) (Figure 3E). The full volume of thymocyte nuclei were analyzed by confocal microscopy. The association of a γH2A.X focus with the *Tcra* locus specifically in DP thymocyte reflects ongoing TCRα rearrangements (Figure 3F). It has been previously described that sorted DP thymocytes from WT mice show around 40% of cells with monoallelic *Tcra*-γH2A.X association, thus performing V(D)J recombination on one TCRα allele; and 10% of cells with biallelic *Tcra*-γH2A.X association, rearranging the two alleles at the same time (38, 39). Defects in the V(D)J recombination machinery lead to various phenotypes. *Atm*^−/−^ and *RAG2*^*c*/*c*^ thymocytes show a “recombination” defect, with a normal frequency of monoallelic *Tcra*-γH2A.X association but a severely increased biallelic association (39) and association of both *Tcra*-γH2A.X and *Igh*-γH2A.X in individual DP thymocytes (38). This misregulated V(D)J recombination with multiple simultaneous RAG cleavages is associated with early T-lymphoma of DP origin in *Atm*^−/−^ (19, 40) and *RAG2*^*c*/*c*^ mice crossed on *Trp53*^−/−^ (12). On the other hand, *53BP1*^−/−^ mice show a “DNA repair” defect, with severely increased monoallelic *Tcra*-γH2A.X association and normal biallelic association, meaning that while the regulation of safe monoallelic RAG-mediated cleavage is efficient, repair of the RAG-mediated DSB is delayed (38).

In this work, WT thymocytes showed 40.2% of monoallelic *Tcra*-γH2A.X association, and 9.2% of biallelic association (Figure 3G) as previously described (38, 39). In contrast, DP thymocytes from 2 independent *Xlf* ^−/−^ mice showed a modest but statistically significant increase in monoallelic *Tcra*-γH2A.X association (47.6 and 47.0% vs. 40.2%, *p* = 0.004 and *p* = 0.006), thus recapitulating a V(D)J “DNA repair” defect to some extent. Biallelic association was only very slightly and not significantly increased, with 11.3 and 11.4% in *Xlf* ^−/−^ cells compared to 9.2% in WT thymocytes. These results suggest that a weakened DNA repair function during TCRα rearrangements in *Xlf* ^−/−^ DP thymocytes could be responsible for the chronic P53 response in these cells, resulting in their reduced viability, thus explaining the immune phenotype.

### Delayed β-Selection in *Xlf*^−/−^ Thymus

Having proposed that subefficient V(D)J recombination, characterized by the slower repair of RAG1/2-mediated DSBs, may participate in the thymocyte phenotype seen in *Xlf* ^−/−^ mice, we revisited the impact of Xlf deficiency on the development of B- and T-lymphocytes *in vivo* using dedicated and sensitive markers. Indeed, if V(D)J recombination is not fully efficient in *Xlf* ^−/−^ mice, it should not only impact TCRα rearrangements but also other *Tcr* and *Ig* loci to some extent.

In the thymus, during the CD4-CD8- DN development stage prior to TCRα rearrangement at the DP stage, V(D)J recombination initiates at the *Tcrb* locus with Dβ-Jβ recombination at DN2 to DN3 transition. The DN3 (CD44- CD25+) stage can be further divided according to the expression of CD28. Complete Vβ-DβJβ recombination occurs during the DN3A (CD44- CD25+ CD28-) stage, followed, if the rearrangement is productive, by the acquisition of the CD28 surface molecule (CD44- CD25+ CD28+, DN3B) and the TCRβ selection induced transition into the DN4 stage (10, 41). Thus, T-cell development first relies on V(D)J recombination efficiency at the *Tcrb* locus during the DN3A stage, and severe V(D)J recombination defects lead to a complete arrest of thymocyte development at this stage *in vivo*, as described in *Xlf*^−/−^ *PAXX*^−/−^ DKO mice for example (27).

**Figure 4:**
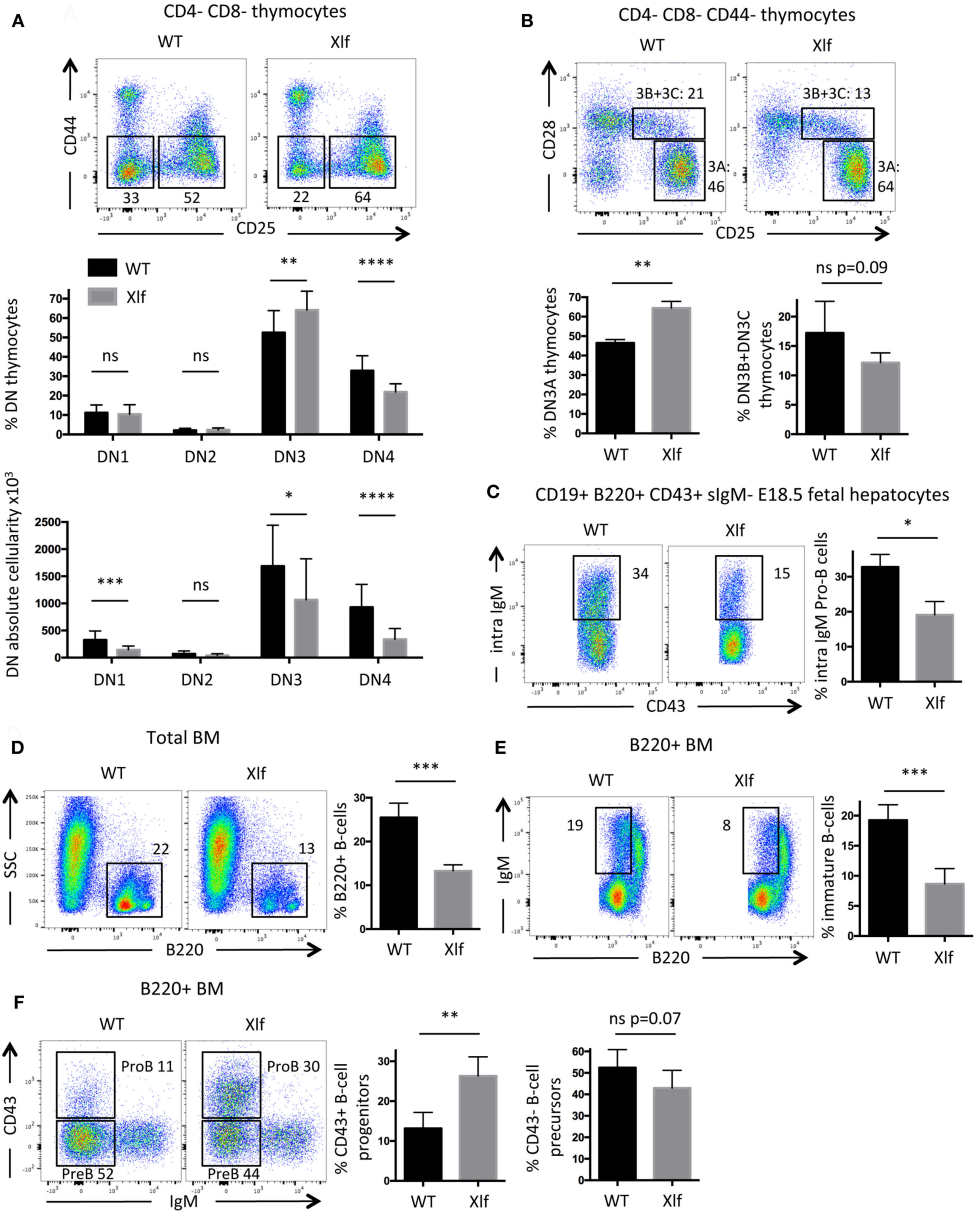
Partial developmental arrest of T- and B-lymphocytes in *Xlf*^−/−^ mice. **(A)** Immunostaining and quantification of CD4-CD8- thymocyte populations. DN3 stage is defined as CD44-CD25+ thymocytes and DN4 stage is defined as CD44-CD25- thymocytes. **(B)** Immunostaining and quantification of CD4-CD8-CD44- thymocyte populations. DN3A stage is defined as CD25+CD28- thymocytes, during which thymocytes undergo V(D)J recombination at *Tcrb* locus. **(C)** Immunostaining and quantification of intracellular IgM expression in E18.5 fetal liver pro-B cells. **(D)** Immunostaining and quantification of B220+ total B-cells in adult bone marrow (BM). **(E)** Immunostaining and quantification of immature B-cells in adult B220+ BM. Immature B-cells stage is defined as B220^low^ IgM+ stage, which results from V(D)J recombination at *IgH* locus in Pro-B cells. **(F)** Immunostaining and quantification of B220+ CD43+ IgM- progenitors B-cells in adult BM. *P*-values were calculated using non-parametric Mann-Whitney tests **(A–F)**.

The commitment of *Xlf* ^−/−^ DN thymocytes to the DN3 stage was not affected, with normal proportions of DN1 and DN2 subsets (Figure 4A). In contrast, *Xlf* ^−/−^ thymocytes demonstrated a statistically significant increase in the DN3 subset (64.1% in *Xlf* ^−/−^ vs. 52.5% in WT, *p* = 0.009) (Figure 4A), attesting for a development delay at this stage. As a consequence, fewer cells proliferated through the DN4 stage (21.9% in *Xlf* ^−/−^ vs. 32.9% in WT, *p* < 0.0001) (Figure 4A). More precisely, *Xlf* ^−/−^ thymocytes accumulated at the DN3A stage (64.4% in *Xlf* ^−/−^ vs. 46.5% in WT, *p* = 0.008) (Figure 4B). This development delay is an evidence of an altered V(D)J recombination in the DN3A stage, which may be overcome in *Xlf* ^−/−^ mice through the high proliferation during the subsequent β-selection and DN4 stage. Indeed, the proliferation of thymocytes through DN4A-B-C stages (data not shown) and the TCRβ repertoire are not affected in *Xlf* ^−/−^ thymocytes (24). The quantification of the absolute numbers of thymocytes revealed a decrease in the various DN subsets, which suggests an overall decrease in thymocyte fitness, independent of V(D)J recombination, in Xlf mice.

### Delayed Intracellular IgM Expression in *Xlf*^−/−^ Pro-B Cells in Fetal Livers

B-cell lymphopenia is an important feature of *Xlf* ^−/−^ condition both in men and mice (20, 22, 25). We wished to analyze to which extent a suboptimal V(D)J recombination could also participate in this aspect of the *Xlf* ^−/−^ phenotype. To evaluate the efficiency of V(D)J recombination during B-cell development, we analyzed B-lymphocyte differentiation in E18.5 fetal livers. Fetal livers allow the study of Pro-B cells in an environment free of mature B-cells, which leave the liver to populate other tissues after expression of surface IgM (sIgM) while losing the CD43 marker. The CD19+ B220+ sIgM- CD43+ Pro-B cells within the liver undergo V(D)J recombination at the *Igh* locus. When achieved, Pro-B cells express intracellular IgM prior to surface expression, which can be quantified by intracellular staining by flow cytometry (27). We observed a significant decrease of intracellular IgM expression in *Xlf* ^−/−^ Pro-B cells (Figure 4C), with 19.1% of intracellular IgM in *Xlf* ^−/−^ vs. 32.8% in WT (*p* = 0.012). These results attest for a mild B cell development alteration compatible with an impaired rearrangement or expression of the *Igh* locus in these fetuses.

### Impaired B Cell Development in Adult *Xlf*^−/−^ Mice Bone Marrow

To further document whether suboptimal *Igh* rearrangements in Pro-B cells could account for the *Xlf* ^−/−^ driven B cell lymphopenia through the lower generation of newly mature B-cells, we analyzed B-cell development in adult bone marrow. The frequency of mature B-cells in young adult *Xlf* ^−/−^ mice (6–9 weeks) was severely and significantly decreased, with 13.3% of total B220+ cells in *Xlf* ^−/−^ vs. 25.5% in WT (*p* = 0.001) (Figure 4D). Interestingly, this was associated with a mild, yet statistically significant, diminution of B220^low^ sIgM+ immature B-cells (8.7% in *Xlf* ^−/−^ vs. 19.3% in WT, *p* = 0.001) (Figure 4E), suggesting an improper generation of newly immature B-cells. Furthermore, we observed a concomitant moderate, yet significant, increase in B220^low^ CD43+ Pro-B cells (26.3% in *Xlf* ^−/−^ vs. 13.2% in WT, *p* = 0.002) among total B220+ cells (Figure 4F), precisely at the development stage when V(D)J recombination at *Igh* locus occurs. Surprisingly, the frequency of B220+CD43-IgM Pre-B cells was not impacted in Xlf mice. This may result from compensatory events as seen in thymus of Xlf/ATM mice with the presence of DP thymocytes despite the severe V(D)J recombination defect (25). Moreover, a decrease in immature B-cells (Figure 4E) while Pre-B cells are not modified suggests that V(D)J recombination at the light chain locus is also somehow compromised in the absence of Xlf.

Altogether, these analyses of B-cell development both in fetuses and adult mice suggest that, like in the T-cell lineage, suboptimal V(D)J recombination may slightly impact B-lymphocyte development at the Pro-B cell stage, resulting in a moderate decrease generation of mature B-cells. However, like in DP thymocytes, this defect is partially overcome and DNA ends are not left unrepaired, since *Xlf* ^−/−^ *Trp53*^−/−^ mice do not develop Pro-B cells lymphomas.

## Discussion

The alteration of the TCRα repertoire observed in Xlf deficient mice (and humans), with reduced utilization of distal TRAV and TRAJ elements suggested two non-exclusive hypotheses: (1) a decrease of thymocyte lifespan at the DP stage related to the positive selection mechanism or (2) a subefficient V(D)J recombination activity. By introducing the Xlf loss of function on a MHC class I and class II DKO we showed that the molecular events associated with positive selection of T-lymphocytes in the thymus have no appreciable impact on the survival of *Xlf* ^−/−^ thymocytes, their P53 chronic activation, and, ultimately, their TCRα repertoire.

We therefore favor the hypothesis of a suboptimal V(D)J recombination process. According to this hypothesis, both B- and T-lymphocyte development are affected in *Xlf* ^−/−^ mice to some extent, with a moderate developmental delay at stages that involve single- or two- steps rearrangements, such as D-to-J and V-to-DJ rearrangements of *Tcra* and *Igh* in thymus, fetal liver, and bone marrow. Interestingly, the in-depth analysis of B cell maturation in the bone marrow of an Xlf deficient patient revealed that although all the maturation steps from Pro-B to mature B cells were represented arguing against an indispensable role of Xlf for V(D)J in humans, the relative high proportion of CD22+/CD19- Pro-B cells as compared to healthy controls was indicative of a partial block of B cell differentiation compatible with a suboptimal V(D)J recombination efficiency (42).

TCRα rearrangements in DP thymocytes proceed through multiple waves of V(D)J recombination ordered from proximal to distal VαJα rearrangements until the appropriate TCRαβ expressing thymocytes undergo positive selection. In *Xlf* ^−/−^ DP thymocytes, we observed an increase in the number of cells harboring a DNA repair focus (γH2A.X) on one TCRα allele, suggesting a moderate “DNA repair defect” during V(D)J recombination of *Tcra* loci. These subefficient rearrangements would accumulate at each one of these waves, ultimately leading to the described *Xlf* ^−/−^ mouse phenotype, i.e., decreased DP thymocyte lifespan through P53 pathway chronic activation accompanied by a biased TCRα repertoire with the loss of distal VαJα rearrangements.

Interestingly, although skewed TCRα repertoire was primarily associated with thymocyte decreased viability, such as in *RORC* deficient mice and patients (29, 33, 34), a similar bias with loss of distal VαJα rearrangements was more broadly described in various human conditions characterized by hypomorphic mutations in known factors of the V(D)J recombination machinery (i.e., *RAG1, Artemis, DNA Ligase IV, Xlf/Cernunnos, PRKDC* genes) with the newly developed tool PROMIDISα (30). *Xlf* ^−/−^ mice analyses further demonstrated that subefficient V(D)J recombination waves could accumulate and lead to immunodeficiency with impoverished TCRα repertoire. Furthermore, the *Xlf* ^−/−^ phenotype only comes out in patients and mice *in vivo*, and has been hidden in *in vitro* V(D)J assays in v-Abl Pro-B cells in many settings (23, 25).

Although V(D)J recombination is subefficient in mice *in vivo*, DSBs are ultimately repaired since *Xlf* ^−/−^ *Trp53*^−/−^ DKO do not develop T or pro-B cell lymphomas (22) and the immune phenotype is even rescued on a *Trp53*^−/−^ background (24). This phenotype is quite different from that of *53BP1*^−/−^ mice for example, which exhibit a more severe “DNA repair” defect during V(D)J recombination waves at *Tcr*α locus (38). Indeed, *53BP1*^−/−^ mice show severe thymic lymphomas at 2 to 4 months of age with TCRαδ translocations (43). Thus, although V(D)J recombination is subefficient in *Xlf* ^−/−^ DP thymocytes, the DNA broken ends are not left unrepaired. This could be explained by the several DNA repair factors that are compensatory for Xlf defect in V(D)J recombination, such as PAXX, RAG2 C-terminus, MRI, ATM, and H2A.X. The double safe-lock provided by the RAG1/2 post cleavage complex (PCC) and many DDR factors (i.e., PAXX, ATM, H2A.X and MRI) (23, 25–27) on the one hand and the Xlf-XRCC4 filament (7, 8) on the other hand may ensure that DNA broken ends are not left unrepaired.

In Xlf deficient patients and mice, B cell lymphopenia is a severe feature and is associated with Class Switch Recombination defects (25, 44). Furthermore, B-cells decline in bone marrow and in spleen worsen with aging, and is associated with the deterioration of hematopoietic stem cells differentiation potential, which particularly affects all hematopoietic lineages in *Xlf* ^−/−^ aged mice (45). The proliferative failure of *Xlf* ^−/−^ hematopoietic stem cells is related to accumulation of unrepaired DSBs, as exemplified in a model of human induced pluripotent stem cell (46). Here we showed that subefficient V(D)J recombination leads to delayed generation of new mature B-cells. Subefficient *Igh* rearrangements could have additive effects with moderate Class Switch Recombination and hematopoietic stem cell defects, which ultimately lead to the premature loss of mature B-cells in adult *Xlf* ^−/−^ mice and Xlf/Cernunnos deficient patients.

Lastly, one cannot exclude an additional role of Xlf in V(D)J recombination, beyond its function during the DNA repair final step. In Xlf deficient patients, coming with TCRα repertoire bias, the Ig and TCRδ repertoires are strongly impoverished because of a specific defect in N-nucleotide addition by the TdT polymerase in productive and unproductive *Ig* and TCR rearrangements (35). This leads to the synthesis of overall one to three amino acids shorter Ig or TCR CDR3 regions. This lower junction diversity likely induces a poorer antigen recognition potential of *Xlf* ^−/−^ lymphocytes (35). Whether the *Xlf* ^−/−^ subefficient V(D)J recombination *in vivo* is linked to a defect in the random nucleotide incorporation by the TdT after Artemis exonuclease activity, leading to a possible delayed synthesis of ligatable DNA ends is an interesting possibility. However this hypothesis would not explain the proliferation delay we observed at the DN3A subset in the thymus, while IJspeert et al. do not show any N-nucleotide addition defect in TCRβ rearrangements.

## Materials and Methods

### Mice

*Xlf*^−/−^ mice were previously described (24). B6.129-B2m tm1Unc.H2-Ab1 tm1Doi /DoiOrl mice with targeted deletion of *MHC-classI (*β*2m)* and *MHC-classII (A*β*)* genes (31) were obtained from TAAM-CDTA Orléans. All animals were maintained in a specific pathogen-free environment. Analyses were performed on *Xlf*^−/−^ *, MHC class I*^−/−^ *MHC class II*^−/−^ DKO and littermate controls on a mixed B6/N background. All experiments and procedures were performed in compliance with the French Ministry of Agriculture's regulations for animal experiments (act 87847, 19 October 1987; modified in May 2001). These studies did not require review and approval by a local ethics committee.

### Flow Cytometry Analysis of Cell Populations

Cell phenotyping from 6 to 9 week old mice was performed on thymus and bone marrow after short hemolysis according to standard protocols by seven-color fluorescence analysis. The following antibodies were used: CD4, CD8, CD25, CD28, CD44, CD69, B220, CD19, and IgM (all from Sony Biotechnology, using respectively PECy7, FITC, PerCPCy5.5, PE, BV510, APC, PE, PECy7, APC fluorophores). Intracellular IgM expression in E18.5 fetal liver cells was performed as previously described (27). The following antibodies were used: CD19, B220, CD43, IgM for extracellular staining followed by cell fixation and permeabilization (Invitrogen) and intracellular IgM staining (all from Sony Biotechnology, using respectively PECy7, BV605, PE, FITC, and APC). Cells were recorded by fluorescence-activated cell sorting (FACS) LSRFortessa X-20 immediately after incubation with Sytox Blue (Life Technologies) to exclude dead cells (except for fetal liver), and analyses were performed with FlowJo 10 software.

### Thymocyte Survival Assay

*Ex vivo* thymocyte survival assay was performed as previously described (24) with minor modifications. Single-cell suspensions were obtained from the thymus and cultured at 3.10^5^ cells in 200 μL in Dulbecco's modified Eagle's medium glutaMAX (DMEM), 10% heat inactivated fetal bovine serum, 1 mM sodium pyruvate, 1X MEM NEAA (Life Technologies), and 50 μM 2-Mercaptoethanol (Life Technologies). Apoptosis was scored after 20 h culture by FACS after labeling with Annexin V and 7-aminoactinomycin D (7AAD) (apoptosis detection kit; BD Pharmingen).

### Quantitative Real-Time RT-PCR Analysis

TaqMan PCR was performed on triplicates of 8 ng of reverse-transcribed RNA from freshly dissected total thymus, as previously described (24) with minor modifications, using predesigned primer and probe sets from Applied Biosystems [Mm01303209_m1 for mouse *Cdkn1a* or P21 exons 1 and 2; Mm00432050_m1 for mouse *Bax* exons 4 and 5; Mm00519268_m1 for mouse *Bbc3*/*PUMA* exons 3 and 4; Mm 99999915_g1 for glyceraldehyde-3-phosphate dehydrogenase (*GAPDH*)]. mRNA expression levels were calculated with ViiA 7 Real-Time PCR System v1.1 (Applied Biosystems). GAPDH was used for normalization of expression, and RNA from WT littermates was used as the calibrator. The relative amounts of mRNA in samples were determined using the 2-ΔΔ*CT* method, where ΔΔ*C*_*T*_is the difference between Δ*C*_*T*_(*C*_*T*_target-*C*_*T*_GAPDH) sample and Δ*C*_*T*_(*C*_*T*_target-*C*_*T*_GAPDH) calibrator. Final results were expressed as *n*-fold differences in target gene expression for tested samples compared with the mean expression value for the controls.

### Analysis of Thymic TCRα Repertoire

Comprehensive TCRα repertoire analyses were performed by 5′ Rapid amplification of complementary DNA (cDNA) ends (5′RACE) PCR/NGS (switching mechanism at the 5′end of the RNA transcript, SMARTα) from total thymus RNA as previously described (24). Five hundred base-pair PCR products were gel purified and processed for single-molecule Illumina sequencing. Sequencing data were analyzed with LymAnalyzer (47) to retrieve unique CDR3 clonotypes and determine T cells receptor alpha variable (TRAV) and T cell receptor alpha junction (TRAJ) gene segments. Output LymAnalyzer files were transformed with R to generate FlowJo9 compatible files with genomic coordinates of the various TRAV and TRAJ pairs. Frequencies of TRAV and TRAJ usage were implemented in PCA analyses using the *PCA()* and *HCPC()* functions of the FactomineR package, respectively [http://factominer.free.fr/ (Le S. Journal of Statistical Software 2008; 25:1-18).] (48) and graphics were generated using the Factoextra R package [http://www.sthda.com/english/rpkgs/factoextra (Kassambara A. Practical guide to cluster analysis in R: STHDA, http://sthda.com; 2017)].

### Immunofluorescence

Thymocytes from 6 to 9 week old mice were dropped on poly-L-lysine coated coverslips. Cells were fixed with 2% paraformaldehyde/1x PBS for 12 min at RT, rinse 3 times in PBS, permeabilized in 0.4% Triton X-100/1x PBS for 5 min on ice, rinse 3 times in PBS, blocked in 2.5% BSA 10% Goat serum 0.1% Tween20/1xPBS for 30 min at RT. Immuno-staining was performed for 60 min in blocking buffer with Anti-phosphoS139-Histone-H2A.X Alexa Fluor 488 Conjugate Antibody (Millipore clone JBW301) or Anti-phosphoS2056-DNAPKcs (Abcam ab18192) followed by an appropriate secondary antibody A488 conjugated. Four washes were performed 5 min each in 0.5% BSA 0.1% Tween20/1x PBS. DNA was stained with 4′,6-diamidino-2-phenylindole (DAPI) and mounting in FluorSave (Calbiochem). Full nucleus volume was imaged through 630X total magnification and 0.2 μm Z-stack using a Spinning Disk Confocal Microscope (Zeiss) and analysis was performed with ImageJ software. At least 200 nuclei per coverslip were analyzed.

### Probes

BAC probes RP23-255N13 (3′α *Tcra*) and RP23-304L21 (5′α *Tcra*) were labeled and amplified with Cy3- or Cy5-dUTP by Random Priming (using Bioprime DNA labeling system, Life Technologies ref 18094011). For each coverslip, 1 μg of each random priming product was precipitated and resuspended in 15 μL of hybridation buffer (50% formamide 1x denhart's solution 0.1% SDS 40 mM NaH_2_PO_4_ 10% sulfate dextran/2x SSC), denatured for 7 min at 80°C and pre-annealed in competition with 5 μg of CotI DNA mouse and 40 μg of DNA salmon sperm of precipitation (Life Technologies) for 30 min at 37°C before overnight hybridation with cells at 37°C.

### 3-Dimensional Immuno-DNA-FISH

γH2A.X – *Tcra* Immuno-FISH were performed as previously described (39, 49). In brief, DP CD4+CD8+ thymocytes from 6 to 9 weeks mice were sorted using CD4-PECy7 and CD8-BV421 conjugated antibodies (all from Sony Biotechnology) on a FACS Aria II SORP. 2.10^5^ cells were dropped on a poly-L-lysine coated coverslip. Cells were fixed in 2% paraformaldehyde/1x PBS for 12 min at RT, rinse 3 times in PBS, permeabilized in 0.4% triton X-100/1x PBS for 5 min on ice, rinse 3 times in PBS, and blocked in 2.5% BSA 10% goat serum 0.1% tween-20/1x PBS for 30 min at RT. γH2A.X Immuno-staining was performed for 60 min in blocking buffer with Anti-phosphoS139-Histone-H2A.X Alexa Fluor 488 Conjugate Antibody (Millipore clone JBW301), and washed 4 times in PBS 5 min each. Cells were post-fixed in 2% paraformaldehyde/1x PBS 10 min at RT, rinse 3 times in 2x SSC, post-permeabilized in 0.1 M HCl/0,7% Triton X-100/H_2_O 10 min on ice, rinse 3 times in 2x SSC, denatured in 1.9 M HCl/H_2_O 30 min at RT, and rinse 3 times in ice cold 2x SSC on ice. Endogenous *Tcra* loci were hybridized with BAC probes RP23-255N13-Cy3 (3′α *Tcra*) and RP23-304L21-Cy5 (5′α *Tcra*) overnight in humid chamber at 37°C in hybridation buffer (50% formamide 1x denhart's solution 0.1% SDS 40 mM NaH_2_PO_4_ 10% sulfate dextran/2x SSC). The next day cells were washed 3 times in 50% formamide/2x SSC and 3 times 2x SSC at 37°C 5 min each, the fifth wash containing 4′,6-diamidino-2-phenylindole (DAPI). Finally, cells were quickly washed in H_2_O and coverslip mounted on FluorSave (Calbiochem). Full nucleus volume was imaged through 630X magnification and 0.2 μm Z-stack using a Spinning Disk Confocal Microscope (Zeiss) and analysis was performed with ImageJ software. At least 550 nuclei per coverslip were analyzed.

### Statistical Analysis

Statistical tests were applied to combined data sets from repeated experiments. Depending on the experiment, Non-Parametric Mann-Whitney test, Non-Parametric Kruskall-Wallis with Dunn's correction test, or two-tail Fisher's exact test, were performed under Prism v6 software with α-risk = 0.05 *P*-values were taken to be significant: ^*^significant 0.05 ≤ *P* < 0.01; ^**^very significant 0.01 ≤ *P* < 0.001; ^***^highly significant 0.001 ≤ *P* < 0.0001; ^****^highly significant *P* ≤ 0.0001.

## Data Availability

The datasets generated for this study are available on request to the corresponding author.

## Author Contributions

J-PdV conceived the project. BR, VA, and JC planned and performed the experiments. BR and J-PdV co-wrote the manuscript. JC revised the manuscript. All the authors agreed to the publication of this manuscript.

### Conflict of Interest Statement

The authors declare that the research was conducted in the absence of any commercial or financial relationships that could be construed as a potential conflict of interest.
